# Impact of measurement noise on escaping saddles in variational quantum algorithms

**DOI:** 10.1038/s41598-026-40123-3

**Published:** 2026-02-17

**Authors:** Eriko Kaminishi, Takashi Mori, Michihiko Sugawara, Naoki Yamamoto

**Affiliations:** 1 Quantum Computing Center, Keio University, 3-14-1 Hiyoshi, Kohoku-ku, Yokohama, Kanagawa 223-8522 Japan; 2 Department of Physics, Keio University, 3-14-1 Hiyoshi, Kohoku-ku, Yokohama, 223-8522 Japan; 3 Department of Applied Physics and Physico-Informatics, Keio University, 3-14-1 Hiyoshi, Kohoku-ku, Yokohama, 223-8522 Kanagawa Japan

**Keywords:** Information theory and computation, Quantum physics, Statistical physics, thermodynamics and nonlinear dynamics

## Abstract

Stochastic gradient descent (SGD) is a widely used optimization technique in classical machine learning and the Variational Quantum Eigensolver (VQE). In VQE implementations on quantum hardware, measurement shot noise is inevitable. We analyze how this noise affects optimization dynamics, especially escape from saddle points in non-convex loss landscapes. Our simulations show that the escape time scales as a power law with respect to $\eta /N_s$, where $\eta$ is the learning rate and $N_s$ is the number of measurements. Through SGD analysis, we provide theoretical insight into how measurement noise facilitates escape. In particular, we demonstrate that a continuous-time approximation via stochastic differential equations (SDE) accurately captures the transient escape dynamics. This suggests that $\eta /N_s$ represents effective noise strength, indicating that increasing $\eta$ or decreasing $N_s$ has similar effects. While concerns exist about the SDE’s validity in stationary regimes, our findings clarify its applicability to transient behavior. Our work improves understanding of the role of measurement noise in VQE optimization.

## Introduction

Quantum computers are expected to potentially impact on various fields, including finance, quantum chemistry, optimization problems, and machine learning^1^. So far, research has primarily focused on quantum algorithms for NISQ devices, such as Variational Quantum Algorithms (VQAs)^2^, yielding significant progress. The most extensively studied algorithm among VQAs is the Variational Quantum Eigensolver (VQE)^3,4^, designed to compute a given Hamiltonian’s ground state energy. Meanwhile, the performance of NISQ devices has been improving rapidly, and in recent years, the realization of early fault-tolerant quantum computers (early FTQC) has also come into view^5,6^.While VQE is a NISQ algorithm, recent works have shown its applicability in FTQC environments. For example,VQE can be used for preoptimization to accelerate FTQC^7,8^, implementing Fault-Tolerant VQE (FT-VQE) to maintain optimization performance under Clifford+T gate constraints^9^ and leveraging pre-trained parameters from NISQ devices as efficient initial guesses for larger-scale fault-tolerant circuits^10,11^.

In VQE, the quantum state $\vert \psi ({\boldsymbol{\theta }})\rangle$ is parameterized by a set of parameters denoted by ${\boldsymbol{\theta }}$, and these parameters are optimized by minimizing the expectation value of the Hamiltonian, $L({\boldsymbol{\theta }})=\langle \psi ({\boldsymbol{\theta }})|H|\psi ({\boldsymbol{\theta }})\rangle$. The optimization is typically performed using gradient descent (GD), where the parameters ${\boldsymbol{\theta }}$ are updated iteratively based on the gradient $g({\boldsymbol{\theta }})=\nabla L({\boldsymbol{\theta }})$ of the loss function. Although gradient-free optimizers have also been proposed^12–15^, this work focuses on gradient-based methods for their efficiency in VQA.

In practice, due to a finite number of quantum measurements (shots), exact gradient computation is not feasible, leading to the introduction of stochastic noise in the gradient estimator $\hat{g}({\boldsymbol{\theta }})$. Consequently, the optimization process becomes a Stochastic Gradient Descent (SGD)^16–19^, where parameters are updated according to ${\boldsymbol{\theta }}^{(k+1)}={\boldsymbol{\theta }}^{(k)}-\eta \hat{g} ({\boldsymbol{\theta }}^{(k)})$, with $\eta>0$ being the learning rate and $\hat{g}({\boldsymbol{\theta }})$ representing the noisy gradient^19^. Despite the introduction of noise, stochastic noise in SGD has been shown to aid in escaping saddle points and local minima, accelerate convergence, and improve generalization performance^20^. Harrow and Napp^16^ demonstrated that SGD can achieve faster convergence with lower computational cost compared to zeroth-order optimization methods in the context of VQA.

One of the key challenges in VQE optimization is escaping from saddle points and local minima, which often impede convergence and lead to suboptimal solutions^21^. The noise introduced by finite-shot measurements, however, plays a crucial role in helping to escape these saddle points, akin to what has been observed in classical optimization. However, understanding the precise dynamics of how measurement noise facilitates this escape remains an open question. While some recent works, such as the “SantaQlaus” approach^22^, hasfocused on exploiting measurement noise for better optimization, many fundamental questions remain. For example, the notorious “barren plateau” problem^23–26^, where the optimization landscape becomes nearly flat, making gradient-based optimization difficult in large-scale quantum systems. Strategies such as shallow circuits and local observables have been proposed to mitigate this issue^27–33^, but more research is needed to fully address it.

Although advances in noise mitigation strategies such as zero-noise extrapolation and other error-correction protocols^34–42^ may eventually suppress or even eliminate gate-level and circuit-level noise in FTQC, measurement noise arising from finite measurement shots will persist. Indeed, measurement noise stems from the intrinsic probabilistic nature of quantum measurements and thus cannot be fully eradicated by fault-tolerant methods. Since measurement noise remains unavoidable, optimization strategies for quantum algorithms need to account for its impact not only in the NISQ era but also in the FTQC era. Particularly, research analyzing the impact of measurement noise in VQE is expected to provide valuable insights for reducing measurement costs and improving optimization performance in FTQC environments. In particular, the insights gained here on saddle-point escape under measurement noise can inform both NISQ-era implementations and future fault-tolerant protocols that must still contend with sampling-induced fluctuations.

A critical aspect of understanding SGD in VQE is the relationship between discrete-time dynamics and their continuous-time approximations. Recent efforts to leverage stochastic differential calculus in the analysis of SGD have investigated how stochastic differential equations (SDEs) can be employed to describe the behavior of SGD^43–49^. These continuous-time frameworks provide analytical insights that are often hard to guess from discrete-time analyses. However, the validity and limitations of these approximations, especially in the context of quantum algorithms like VQE, have not been thoroughly investigated.

In this paper, we provide a quantitative analysis of saddle point escape under finite measurement noise in VQE. By analyzing the dynamics of SGD, we offer analytical support for the numerically observed power-law scaling of escape times with respect to $\eta /N_s$, where $\eta$ is the learning rate and $N_s$ is the number of measurements. Specifically, we demonstrate that the continuous-time approximation of discrete-time SGD, described by a SDE, accurately captures the transient dynamics involved in escaping saddle points. This indicates that $\eta /N_s$ can be interpreted as the effective strength of measurement noise, suggesting an equivalence between increasing the learning rate and decreasing the number of measurements.

Furthermore, we emphasize that the continuous-time approximation via SDEs offers valuable insights into the relationship between noise, learning rate, and measurement counts, helping to guide the choice of these parameters in VQE and other VQAs. While previous concerns have been raised about the validity of the continuous-time SDE due to subtleties in its derivation, our findings clarify its applicability to transient dynamics, even though it does not correctly capture long-time fluctuations in a stationary state.

The rest of this paper is organized as follows. Section Measurement noise in variational quantum algorithms discusses the properties of measurement noise in VQE. Section Validity of Continuous time approximation for SGD in VQE introduces the SDE framework as a continuous-time approximation of finite-shot SGD, discussing some subtleties involved in this approximation. Section Effect of measurement noise for the optimization dynamics provides a numerical investigation of escape times from saddle points and examines how these times depend on the learning rate $\eta$ and the number of measurement shots $N_s$. Section Dependence on Parameter Regimes: 6-Qubit Results examines the dependence of noise-assisted escape on parameter regimes by extending the analysis to a 6-qubit system. Finally, Section Conclusion outlines future research directions for optimizing VQE performance.

## Measurement noise in variational quantum algorithms

### Setup of gradient-based methods in VQE

Suppose that we want to obtain the ground state and its energy of a given Hamiltonian *H* of *n* qubits. In VQE, we construct an ansatz state $\vert \psi ({\boldsymbol{\theta }})\rangle =U({\boldsymbol{\theta }})\vert \psi _0\rangle$, where $U({\boldsymbol{\theta }})$ is an unitary operator corresponding to an entire quantum circuit parameterized by ${\boldsymbol{\theta }}$. We then try to obtain the ground state of *H* by minimizing the loss function $L({\boldsymbol{\theta }})=\langle \psi ({\boldsymbol{\theta }})|H|\psi ({\boldsymbol{\theta }})\rangle$. As is briefly mentioned in Introduction, in the GD, the parameters are optimized by repeating the following update:

1 $$\begin{aligned} {\boldsymbol{\theta }}^{(k+1)}={\boldsymbol{\theta }}^{(k)}-\eta \nabla L({\boldsymbol{\theta }}^{(k)}), \end{aligned}$$

where $\eta>0$ is the learning rate. However, in principle, infinitely many quantum measurements are necessary to exactly obtain $\nabla L({\boldsymbol{\theta }})$. In practice, it is impossible to perform quantum measurement infinitely many times: what we can do is approximately estimating $\nabla L({\boldsymbol{\theta }})$ by performing a finite number $N_s$ of quantum measurements. In essence, the gradient calculation is performed by using shot averages. Because of the stochastic nature of the measurement outcome in quantum theory, the estimator $\hat{g}({\boldsymbol{\theta }})$ of $\nabla L({\boldsymbol{\theta }})$ is a stochastic variable. In the SGD, we optimize ${\boldsymbol{\theta }}$ by using this stochastic estimator:

2 $$\begin{aligned} {\boldsymbol{\theta }}^{(k+1)}={\boldsymbol{\theta }}^{(k)}-\eta \hat{g}({\boldsymbol{\theta }}^{(k)}). \end{aligned}$$

### Noise structure of SGD for VQE

There are several ways to evaluate gradients on a quantum circuit. Among them, we focus on the parameter-shift rule^50–52^.

Before introducing the parameter-shift rule, we first discuss a naive method called the finite difference method. The finite difference method gives an approximation of the derivative, whose quality depends on $\epsilon$. It approximates the gradient as

3 $$\begin{aligned} \frac{\partial L({\boldsymbol{\theta }})}{\partial {\boldsymbol{\theta }}_i}\approx \frac{L({\boldsymbol{\theta }} + \epsilon {\boldsymbol{e}}_i) - L({\boldsymbol{\theta }} - \epsilon {\boldsymbol{e}}_i)}{2\epsilon }, \end{aligned}$$

where ${\boldsymbol{e}}_i$ denotes the unit vector along *i*th direction. In this method, even if we exactly evaluate the right-hand side by performing infinitely many quantum measurements ($N_s\rightarrow \infty$), we still have an error due to a finite value of $\epsilon$. If we choose a too small value of $\epsilon$, the error due to a finite $N_s$ is greatly enhanced. Thus, there is a trade-off between the error due to a finite $\epsilon$ and that due to a finite $N_s$.

The parameter-shift rule is an unbiased estimator of the gradient, meaning that it gives the exact gradient if we could perform infinitely many quantum measurements. A crucial assumption is that the unitary gate $U(\theta )$ is generated by a Hermitian operator *G* that has only two eigenvalues $g_0$ and $g_1$: $U(\theta )=e^{i\theta G}$. Then, the gradient can be computed by using finite shifts of parameters. The derivative of the function $f(\theta )=\langle \psi \vert U(\theta )OU^\dagger (\theta )\vert \psi \rangle$ is then given as follows:

4 $$\begin{aligned} \frac{df(\theta )}{d\theta }=r\left[ f(\theta +\pi /4r)-f(\theta -\pi /4r)\right] , \end{aligned}$$

where $r=(g_1-g_0)/2$. All standard parameterized gates that are used in this work have $r=1/2$. We therefore have

5 $$\begin{aligned} \frac{\partial L({\boldsymbol{\theta }})}{\partial {\boldsymbol{\theta }}_i}= \frac{L({\boldsymbol{\theta }}+\frac{\pi }{2} {\boldsymbol{e}}_i)-L({\boldsymbol{\theta }}-\frac{\pi }{2} {\boldsymbol{e}}_i)}{2}. \end{aligned}$$

Mari et al.^53^ compared finite-difference gradient estimator and the parameter-shift gradient estimator: they tested their predictions by numerical simulations and real quantum experiments. They showed that the error of $\partial L/\partial \theta _i$ in the finite-difference method optimally scales as $N_s^{-1/3}$ by appropriately choosing $\epsilon$ for a given $N_s$, while that of the parameter-shift rule scales as $N_s^{-1/2}$. The parameter-shift rule thus achieves a smaller error with a fixed number of measurements, so that we utilize parameter-shift rule to implement gradient method in quantum algorithms.

When we use the parameter shift rule, a gradient estimator $\hat{g}({\boldsymbol{\theta }})$ contains measurement noise fluctuations. Equation (2) is rewritten as

6 $$\begin{aligned} {\boldsymbol{\theta }}^{(k+1)}={\boldsymbol{\theta }}^{(k)}-\eta \nabla L({\boldsymbol{\theta }}^{(k)})+\eta {\boldsymbol{\xi }}({\boldsymbol{\theta }}^{(k)}), \end{aligned}$$

where ${\boldsymbol{\xi }}({\boldsymbol{\theta }}){:=}-[\hat{g}({\boldsymbol{\theta }})-\nabla L({\boldsymbol{\theta }})]$ is the noise vector. We denote by $\mathbb {E}[\cdot ]$ the average over the infinitely many repetitions of quantum measurements. We then have $\mathbb {E}[{\boldsymbol{\xi }}]=0$.

In Eq. (2), time steps needed for ${\boldsymbol{\theta }}$ to change is proportional to $1/\eta$, during which the total number of measurements is proportional to $N_s/\eta$. When it is large enough, noise can be regarded as Gaussian, owing to the central limit theorem. Thus, for small $\eta /N_s$, noise is considered to be Gaussian, which is characterized by the covariance matrix

7 $$\begin{aligned} C({\boldsymbol{\theta }}){:=}\mathbb {E}[{\boldsymbol{\xi }}({\boldsymbol{\theta }}){\boldsymbol{\xi }}^\top ({\boldsymbol{\theta }})]. \end{aligned}$$

Its matrix element is given by $C_{ij}({\boldsymbol{\theta }})=\mathbb {E}[\xi _i({\boldsymbol{\theta }})\xi _j({\boldsymbol{\theta }})]$.

Now let us calculate the covariance matrix of noise for the parameter-shift rule. Suppose that the Hamiltonian is decomposed as

8 $$\begin{aligned} H=\sum _lh_l, \end{aligned}$$

and quantum measurements are performed $N_s$ times for each $h_k$ separately. Under this setting, by using Eq. (5), $C({\boldsymbol{\theta }})$ is calculated as

9 $$\begin{aligned} C_{ij}({\boldsymbol{\theta }})&=\frac{\delta _{ij}}{4N_s}\sum _k\left( \langle {\boldsymbol{\theta }}^{(i+)}\vert h_k^2\vert {\boldsymbol{\theta }}^{(i+)}\rangle -\langle {\boldsymbol{\theta }}^{(i+)}\vert h_k\vert {\boldsymbol{\theta }}^{(i+)}\rangle ^2\right. \nonumber \\&\left. +\langle {\boldsymbol{\theta }}^{(i-)}\vert h_k^2\vert {\boldsymbol{\theta }}^{(i-)}\rangle -\langle {\boldsymbol{\theta }}^{(i-)}\vert h_k\vert {\boldsymbol{\theta }}^{(i-)}\rangle ^2\right) , \end{aligned}$$

where we used simplified notations ${\boldsymbol{\theta }}^{(i\pm )}{:=}{\boldsymbol{\theta }}\pm (\pi /2){\boldsymbol{e}}_i$.

At this point, we note that when measuring gradients with VQE, each gradient component is evaluated independently. The exact gradient $\nabla L({\boldsymbol{\theta }})$ can be obtained through infinitely many quantum measurements. However, in practice, we can only obtain the stochastic gradient estimator given as an average value over a finite number of quantum measurements. Therefore, the covariance matrix of SGD in VQE has no off-diagonal matrix element: $C_{ij}({\boldsymbol{\theta }})=0$ for any $i\ne j$.

The structure of the covariance matrix of SGD in VQA differs from that in machine learning, where noise stems from the mini-batch sampling of training data. In classical machine learning, it was argued that strong anisotropic nature of the noise covariance matrix, which is associated with nontrivial structure of off-diagonal matrix elements, is beneficial for generalization^54–56^ and for optimization^48,54,57–61^. In contrast, the covariance matrix of SGD for VQE has no off-diagonal elements.

The mini-batch sampling noise in machine learning also has another interesting property: noise strength is correlated with the value of the loss function^61^, and noise can vanish at a global minimum. By contrast, the measurement shot noise does not vanish even if our quantum state $\vert \psi ({\boldsymbol{\theta }})\rangle$ realizes the exact ground state of $\hat{H}$, unless $\hat{H}$ is a frustration-free Hamiltonian, where all the terms $\{h_k\}$ can be simultaneously minimized in the ground state.

In this context, the nature of stochastic noise in VQE is qualitatively different from that in classical machine learning. In classical settings, SGD noise originates from mini-batch sampling and is closely related to generalization properties, overparameterization, and implicit regularization effects. By contrast, in VQE the dominant source of stochasticity arises from a finite number of quantum measurements. As a result, the role of SGD noise in VQE cannot be directly interpreted through the same lens as in classical learning, and its impact on optimization dynamics is still not fully understood. In the following, we therefore focus on clarifying how measurement noise influences escape from saddle points in VQA.

## Validity of continuous time approximation for SGD in VQE

When the parameter update for each iteration is small, which is typically the case when the learning rate $\eta$ is small enough, we can consider the continuous-time approximation^62,63^. When analyzing discrete SGD theoretically, various studies have been conducted using stochastic differential equations (SDE) with a continuous-time approximation^43–49^. The continuous-time approximation is considered to be valid for a small learning rate, but Yaida^64^ has pointed out that the SDE in the continuous-time limit may not reproduce the correct result even in the limit of small learning rate. In particular, steady-state fluctuations in discrete SGD and continuous SDE are in general different. It is therefore important to verify the validity of the continuous-time approximation of SGD in VQE.

**Fig. 1:**
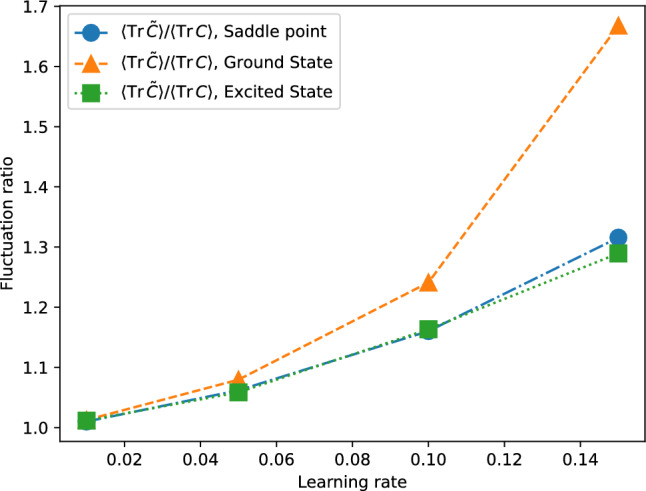
Comparison of $\tilde{C}$ and *C* at (quasi-)stationary state around the ground state, the saddle point same as in Fig. 4, and the excited state same as in Figure 5. We plot the values of $({{\,\mathrm{\textrm{Tr}}\,}}\tilde{C})/({{\,\mathrm{\textrm{Tr}}\,}}C)$ for varying $\eta$ with the noise level $\eta /N_s$ fixed to be 0.0005. The ratio $({{\,\mathrm{\textrm{Tr}}\,}}\tilde{C})/({{\,\mathrm{\textrm{Tr}}\,}}C)$ quantifies the deviation between steady-state fluctuations predicted by the continuous-time SDE and those observed in discrete-time SGD. The behavior of this ratio depends on the location in the loss landscape, reflecting differences in local curvature and measurement statistics near each type of critical point, illustrating that the continuous-time SDE does not fully capture steady-state fluctuations of discrete-time SGD.

In this section, we introduce the SDE framework as an approximation of finite-shot SGD in VQE and discuss the subtleties involved in this approximation, particularly in relation to the learning rate and noise strength. We then analyze the effectiveness of the SDE approach in capturing the escape dynamics from saddle points, as observed in our numerical simulations. Furthermore, we explore the limitations of the continuous-time approximation, especially in describing steady-state fluctuations. From our numerical calculations presented in Figure 1, it turns out that the continuous-time SDE reproduces steady-state fluctuations of the discrete-time SGD for sufficiently small learning rates. Later, in Section Effect of measurement noise for the optimization dynamics, we also see that the continuous-time SDE gives correct results on the escape time from a saddle even beyond the apparent applicability of the SDE, i.e., even for relatively large learning rates. Our findings suggest that while the continuous-time limit may not accurately describe the full spectrum of VQE dynamics for non-small learning rates, it is highly effective in characterizing transient dynamics.

### Stochastic differential equation for SGD in VQE

For small enough constant learning rate $\eta$, Eq. (2) is regarded as a discrete update (i.e. the Euler-Maruyama discretization) of the following SDE by considering $\eta$ as an infinitesimal time step: $\eta =dt$^43,62,63^:

10 $$\begin{aligned} d{\boldsymbol{\theta }}_t=-{\boldsymbol{\nabla }}L({\boldsymbol{\theta }}_t)dt +\sqrt{\eta C({\boldsymbol{\theta }}_t)}\cdot dW_t, \end{aligned}$$

where $W_t$ is a Wiener process, and $dW_t=\mathcal {N}(0,I_pdt)$ with $I_n$ being the *n*-by-*n* identity matrix. The product $\sqrt{\eta C({\boldsymbol{\theta }}_t)}\cdot dW_t$ is interpreted as Itô. The continuous time variable *t* corresponds to $\eta k$ in the discrete SGD.

A tricky point is that we have $\eta$ dependence of the noise term in Eq. (10). This remaining $\eta$ should be treated as a finite value, although we take the limit of $\eta \rightarrow +0$ in the derivation of Eq. (10). Such a treatment is rather heuristic and inconsistent from the mathematical viewpoint^64^. Despite this, our results demonstrate that the SDE correctly reproduces transient behavior, such as the escape from saddle points. These behaviors will be discussed in detail with reference to numerical results later in Section Effect of measurement noise for the optimization dynamics(see Figs. 4 and 5). This implies that while the continuous-time SDE may not be fully accurate for steady-state behavior, it remains highly effective in describing transient dynamics.

An immediate implication of Eq. (9) and Eq. (10) is that the effect of noise should appear as a function of $v{:=}\sqrt{\eta /N_s}$. Reducing $N_s$ has the same effect as increasing $\eta$. Therefore, as long as Eq. (10) is valid, the escape time must be written as a function of *v*, which is a nontrivial scaling relation predicted by the SDE.

### Properties of steady-state fluctuations in VQA

Yaida^64^ derived “fluctuation-dissipation relations” for the SGD algorithm. These relations can be used to adaptively set training schedule and be used to efficiently extract information pertaining to a loss-function landscape such as the magnitudes of its Hessian. Using the stationarity assumption that the *k*th and $(k+1)$th steps of the optimization process have the same probability distribution, the following relation is derived^64^:

11 $$\begin{aligned} \langle ({{\boldsymbol{\theta }}}\cdot \nabla L({{\boldsymbol{\theta }}}))\rangle =\frac{\eta }{2}\langle {{\,\mathrm{\textrm{Tr}}\,}}\tilde{C}({\boldsymbol{\theta }})\rangle , \end{aligned}$$

where stationary-state average is defined as

12 $$\begin{aligned} \langle O({{\boldsymbol{\theta }}})\rangle = \int d{\boldsymbol{\theta }}p_{ss}({{\boldsymbol{\theta }}})O({{\boldsymbol{\theta }}}) \end{aligned}$$

for an arbitrary observable $O({{\boldsymbol{\theta }}})$ and for an arbitrary stationary distribution $p_{ss}({\boldsymbol{\theta }})$, which satisfies $p_{ss}({\boldsymbol{\theta }}^{(k)})=p_{ss}({\boldsymbol{\theta }}^{(k+1)})$. Equation 11 is approximately valid even when the stationarity is not exact but almost satisfied: $p_{ss}({\boldsymbol{\theta }}^{(k)})\approx p_{ss}({\boldsymbol{\theta }}^{(k+1)})$, e.g. when the parameters are trapped by a saddle for a long time. Throughout the remainder of this manuscript, we use angle brackets $\langle \cdot \rangle$ to indicate an average taken with respect to a quasi-stationary distribution $p_{ss}(\theta )$. In other words, even if the distribution of parameters is not strictly stationary, it may remain nearly unchanged over a certain timescale (for instance, when the parameters are stuck near a saddle point). By contrast, we reserve the notation $\mathbb {E}[\cdot ]$ exclusively for the average over the infinitely many repetitions of quantum measurements. We emphasize this distinction because fluctuations in $\theta$ arise from the optimization trajectory itself, whereas the measurement noise described by $\mathbb {E}[\cdot ]$ concerns the quantum measurement outcomes for a fixed parameter $\theta$.

Following Yaida^64^, we define $p_{ss}({\boldsymbol{\theta }})$ as the stationary distribution that satisfies the condition

13 $$\begin{aligned} \int d{\boldsymbol{\theta }}p_{ss}({{\boldsymbol{\theta }}})O({{\boldsymbol{\theta }}})=\int d{\boldsymbol{\theta }}p_{ss}({{\boldsymbol{\theta }}})\mathbb {E}[O({\boldsymbol{\theta }}-\eta \hat{g}({\boldsymbol{\theta }}))]. \end{aligned}$$

This relation expresses that the probability distribution of the parameters remains unchanged under the SGD update Eq. (2). It provides a discrete-time analogue to the stationary distribution in the Fokker-Planck formalism.

A remarkable point of Eq. (11) is that $\tilde{C}$ in the right hand side is *not* the noise covariance matrix given by Eq. (7), but the second-order moment matrix of the gradient, which is defined as

14 $$\begin{aligned} \tilde{C}_{ij}({\boldsymbol{\theta }}) {:=}\mathbb {E}[\hat{g}_i({{\boldsymbol{\theta }}})\hat{g}_j({{\boldsymbol{\theta }}})]. \end{aligned}$$

If we derive an analogous relation for the continuous-time SDE, i.e., Eq. (10), we would obtain the equation in which $\tilde{C}({\boldsymbol{\theta }})$ is replaced by $C({\boldsymbol{\theta }})$ in Eq. (11). This difference stems from the subtlety of the derivation of the SDE explained in Section Stochastic differential equation for SGD in VQE. The above fact suggests that the continuous-time SDE is not valid when $C({\boldsymbol{\theta }})$ significantly differs from $\tilde{C}({\boldsymbol{\theta }})$.

Figure 1 shows the ratio of $\langle C({\boldsymbol{\theta }})\rangle$ to $\langle \tilde{C}({\boldsymbol{\theta }})\rangle$ as a function of the learning rate $\eta$ at the ground state (a global minimum of the loss function), a saddle point that is discussed in Section Escaping from saddle points, and an excited state that is discussed in Section Escaping from an excited state. We compute $\langle C({\boldsymbol{\theta }})\rangle$ and $\langle \tilde{C}({\boldsymbol{\theta }})\rangle$ by considering a time average instead of the average over a (quasi-)stationary distribution $p_{ss}$:

15 $$\begin{aligned} \langle O({\boldsymbol{\theta }})\rangle \approx \frac{1}{n}\sum _{k=1}^n O({\boldsymbol{\theta }}^{(k)}), \end{aligned}$$

where ${\boldsymbol{\theta }}^{(k)}$ is generated by Eq. (2) starting near a critical point (the ground state, a saddle point, or an excited state) and the time step *n* is chosen as large as possible but smaller than the escape time step. Figure 1 shows that, by changing $\eta$ with $\eta /N_s$ held fixed, *C* and $\tilde{C}$ are close to each other for sufficiently small learning rate, but they show strong deviations by increasing $\eta$. In this way, fluctuations at a steady state in the SDE differ from those in the SGD unless the learning rate is small enough.

While these differences in steady-state fluctuations are notable, we will show in Section Effect of measurement noise for the optimization dynamics that they do not substantially impact the escape dynamics. This observation is significant because it demonstrates that even though the continuous-time approximation may not fully capture the behavior of steady-state fluctuations, it remains a powerful tool for accurately describing the transient dynamics in VQE optimization.

## Effect of measurement noise for the optimization dynamics

It has been reported that implementing SGD instead of GD makes the convergence faster^18,19^. Some theorems have shown that noisy update of the parameters can avoid to get stuck at saddle points or local minima and therefore is beneficial for the optimization in the classical machine learning^65,66^ and in VQAs^18^. However, theoretical bounds on the escape time are not tight at all, and hence quantitative studies on the escape time from saddles in VQEs are still lacking.

In this section, we consider SGD implemented in VQEs, and numerically evaluate the escape time from saddle points in Section Escaping from saddle points and from an excited state, which is a marginally stable point of the loss function (i.e. the minimum eigenvalue of the Hessian of the loss function is almost zero), in Section Escaping from an excited state. We will numerically show that the escape time in the SGD algorithm is given as a function of $v=\sqrt{\eta /N_s}$, which is predicted by the continuous-time SDE. Indeed, the escape time follows a power-law scaling with respect to *v*. We also argue that the computational cost in escaping from a saddle also depends on *v* and polynomially decreases with increasing *v*.

In the following, we consider the problem of finding the ground state of the one-dimensional (1D) Heisenberg model as a prototypical example of VQEs. The Hamiltonian is given by

16 $$\begin{aligned} H=\sum _{i=1}^4\left( X_iX_{i+1}+Y_iY_{i+1}+Z_iZ_{i+1}\right) , \end{aligned}$$

where $X_i$, $Y_i$, and $Z_i$ are Pauli operators at site *i*. In performing quantum measurements, we decompose the Hamiltonian as $H=\sum _{l=1}^3h_l$, where

17 $$\begin{aligned} h_1=\sum _{i=1}^4X_iX_{i+1}, \quad h_2=\sum _{i=1}^4 Y_iY_{i+1}, \quad h_3=\sum _{i=1}^4Z_iZ_{i+1}. \end{aligned}$$

That is, in evaluating $L({\boldsymbol{\theta }}\pm \pi /2{\boldsymbol{e}}_i)$ in the parameter-shift rule (see Eq. (5), we perform $N_s$ quantum measurements for each $h_k$ separately.

**Fig. 2:**
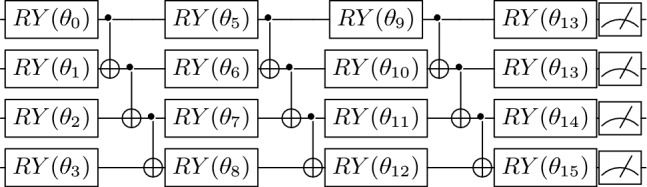
4-qubit RY ansatz for 1D Heisenberg model.

The ansatz is taken as a typical hardware-efficient ansatz that consists of alternating layers of $R_y$ rotations and CNOT entanglements illustrated in Fig. 2.

### Escaping from saddle points

In the high-dimensional loss landscape, it is known that most critical points are saddles^67,68^, and hence it is significant to efficiently escape from saddles in the gradient-based optimization algorithms.

**Fig. 3:**
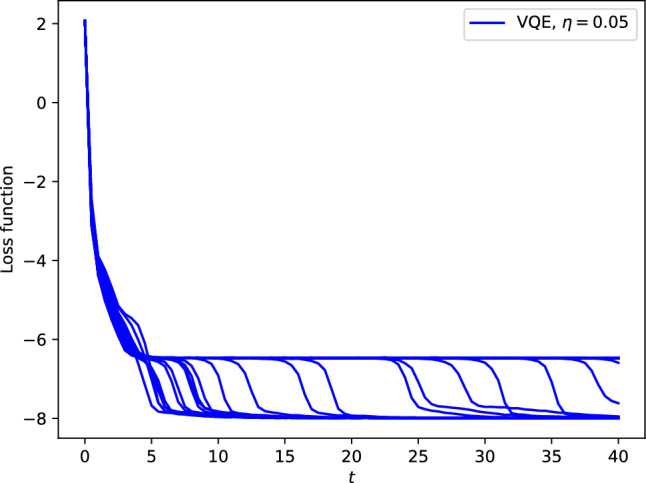
Occurrences of saddle points in the SGD convergence process. We prepare 30 instances starting from same initial condition. Learning rate $\eta$ is 0.05. The number of measurements at each step is 100. In many cases the parameters ${\boldsymbol{\theta }}$ are trapped at a saddle point before converging to the ground state with the energy $-8$.

Figure 3 shows the energy trajectories $\langle {\boldsymbol{\theta }}^{(k)}|\hat{H}|{\boldsymbol{\theta }}^{(k)}\rangle$ against $t=k\eta$ for 30 realizations of the SGD from same randomly chosen initial condition. This initial condition was selected from random initializations that show trapping behavior near a saddle point. The learning rate $\eta$ is 0.05 and the number $N_s$ of measurement shots at each step is 100. As shown, the SGD dynamics often shows a plateau, which is due to a saddle point.

In a plateau of Figure 3, the parameters stay near a saddle point with the energy around $-6.47$. At this saddle point, the Hessian has several positive eigenvalues and a few negative ones, indicating an unstable saddle structure with multiple descending directions in the loss landscape. The explicit eigenvalues are provided in the Supplementary Information.

We refer to this saddle point, which is observed in the convergence trajectories shown in Figure 3, as *Saddle point A* in the following. We measure the escape time from this saddle point. The initial state is chosen as the state at $t=5.4$ generated by the GD in Figure 3, i.e., near the saddle point mentioned above. Starting from this initial state ${\boldsymbol{\theta }}^{(0)}$, we update the parameters ${\boldsymbol{\theta }}^{(k)}$ via Eq. (2). The escape time $t_\textrm{esc}$ is identified as the time $t_\textrm{esc}=\eta k$ at which the energy $L({\boldsymbol{\theta }}^{(k)})$ falls below $-7.0$.

**Fig. 4:**
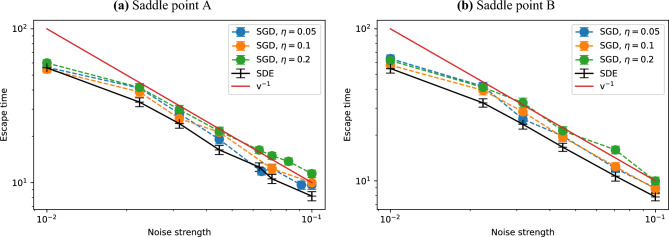
Escape time from saddle points with measurement noise. Panels (a) and (b) correspond to two different saddle points (obtained from different initializations). For both discrete-time SGD and the continuous-time SDE, we prepare 100 instances starting from the same initial condition for each saddle point and compute the escape time averaged over those instances. We find that the escape time is approximately proportional to the inverse of the noise strength.

Figure 4 (a) shows escape times from saddle point A against $\sqrt{\eta /N_s}$, which turns out to measure the noise strength as discussed in Section Validity of Continuous time approximation for SGD in VQE, for different values of $\eta$ (black solid lines in Fig. 4 (a) show the results for the continuous-time stochastic differential equation in Section Validity of Continuous time approximation for SGD in VQE). We prepare 100 instances starting from the same initial condition, and the average escape times, as well as their error bars, are plotted. As intuitively predicted, the escape time decreases as the noise level increases. Remarkably, the escape times for different values of $\eta$ align along a straight line when we plot them against $v=\sqrt{\eta /N_s}$. Furthermore, we find from Fig. 4 (a) that the escape time seems to be inversely proportional to the noise strength,

18 $$\begin{aligned} t_\textrm{esc}\propto v^{-1}. \end{aligned}$$

It should be noted that even the deterministic GD (no noise at all) eventually escapes from the saddle in a finite time, and hence the escape time in the SGD should not diverge in the limit of $v\rightarrow +0$. Therefore, Eq. (18) is valid for not too small values of *v*, and $t_\textrm{esc}$ should be saturated at a finite value for sufficiently small *v*.

We also study the escape from another saddle point. Starting from a different initial state, numerical calculations analogous to those in Figure 3 leads to a different saddle point. We refer to this saddle point as *Saddle point B* in the following.

This saddle point B has an energy around $-6.48$. The Hessian spectrum at saddle point B again contains both positive and negative eigenvalues, confirming the presence of unstable directions. The full list of eigenvalues is given in the Supplementary Information. We evaluated the escape time from saddle point B as shown in Figure 4 (b). When plotted against $v=\sqrt{\eta /N_s}$, the escape times for different values of $\eta$ again exhibit a clear power-law trend. It suggests that the escape times from a saddle point is generally expressed as a function of $v=\sqrt{\eta /N_s}$. Furthermore, we again see that the escape time is inversely proportional to the noise strength, $t_{esc} \propto v^{-1}$.

Here, let us comment on the $\eta$ and $N_s$ dependencies of the overall measurement cost. The measurement cost is defined as the total number of quantum measurements required to escape from the saddle. It is proportional to $N_st_\textrm{esc}/\eta =t_\textrm{esc}/v^2$ (it follows from the fact that $t_\textrm{esc}/\eta$ is the total number of steps to escape the saddle). Since $t_\textrm{esc}$ is a function only of *v*, the measurement cost is also written as a function of *v*. Therefore, for a fixed value of the noise strength *v*, the total number of measurements required to escape is the same regardless of whether the learning rate is increased or decreased. By considering Eq. (18), we find

19 $$\begin{aligned} \text {measurement cost}\propto v^{-3} \end{aligned}$$

for not too small values of *v*. In the limit of $v\rightarrow +0$, $t_\textrm{esc}$ should be finite and hence

20 $$\begin{aligned} \text {measurement cost}\sim v^{-2} \quad (v\rightarrow +0). \end{aligned}$$

### Escaping from an excited state

We find a parameter ${\boldsymbol{\theta }}_\textrm{exc}$ in which $\vert \psi ({\boldsymbol{\theta }}_\textrm{exc})\rangle$ is close to an exact excited state with the energy $-4$. At ${\boldsymbol{\theta }}_\textrm{exc}$, the gradient of the loss function almost vanishes. At this excited-state solution, the Hessian spectrum contains several near-zero eigenvalues, indicating marginally stable flat directions in the loss landscape. The explicit eigenvalues are provided in the Supplementary Information. So ${\boldsymbol{\theta }}={\boldsymbol{\theta }}_\textrm{exc}$ corresponds to a marginally stable point, where the minimum eigenvalue of the Hessian is very close to zero (i.e. the loss landscape has some flat directions).

The escape time $t_\textrm{esc}$ is now defined as time when the energy value falls below $-5.0$.

**Fig. 5:**
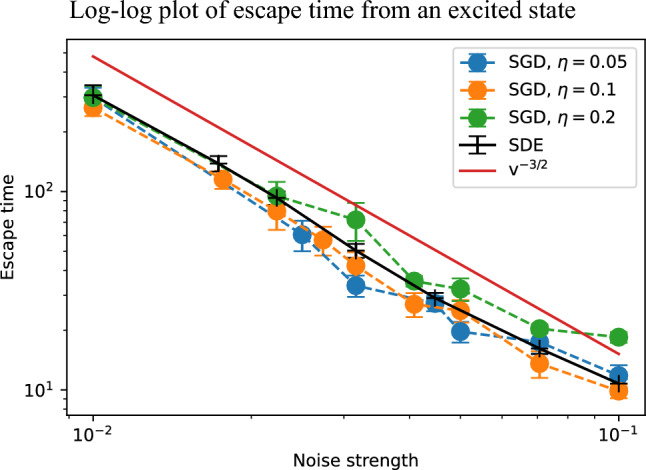
Escape times from an excited state, averaged over 100 instances starting from the same initial condition, for SGD with three different values of $\eta$ and the continuous-time SDE. We find the escape time behaves as $v^{-3/2}$ as indicated by the red line.

Figure 5 shows the escape time from the excited state. We prepare 100 instances starting from the same initial conditions. We also investigate the escape time from an excited state, as depicted in Figure 5. Similarly to Figure 4, when plotted on a log-log scale against $v=\sqrt{\eta /N_s}$, the data align along a straight line, revealing a power-law dependence. This analysis further supports the notion that the escape times from the saddle points and excited state are characterized by a function of the noise strength $\eta /N_s$. Furthermore, we find that the escape time behaves as

21 $$\begin{aligned} t_\textrm{esc}{\propto }v^{-3/2}, \end{aligned}$$

which differs from the scaling in the escape from a saddle (see Eq. (18)).

## Dependence on parameter regimes: 6-qubit results

We investigate how the noise-assisted escape dynamics depend on the parameter regime by extending our analysis to 6-qubit systems. We consider the 6-qubit Heisenberg model with the same Hamiltonian structure as in Section Effect of measurement noise for the optimization dynamics. The ansatz circuit belongs to the same hardware-efficient family used in the 4-qubit analysis, but we examine two different parameter regimes, a reduced-parameter ansatz with 18 parameters, and a highly parameterized ansatz with 48 parameters. As in the previous sections, gradients are evaluated using the parameter-shift rule under a finite number of measurement shots, and SGD dynamics are simulated. Due to the increased computational cost for the 6-qubit system, all escape times reported in this section are averaged over 30 independent realizations. The escape time is defined in the same manner as in Section Effect of measurement noise for the optimization dynamics, namely as the time at which the energy falls below a fixed threshold chosen to be well separated from the saddle-point energy.

**Fig. 6:**
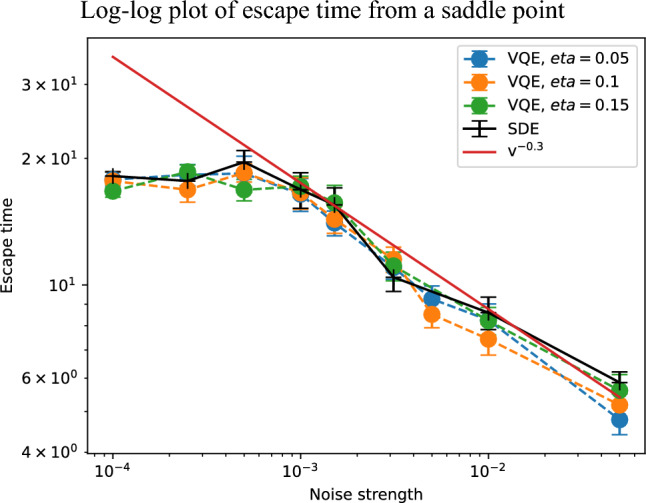
Escape times from a saddle point in the 6-qubit ansatz with 18 parameters, averaged over 30 instances starting from the same initial condition, for SGD with three different values of $\eta$ and the continuous-time SDE. We find the escape time behaves as $v^{-0.3}$ as indicated by the red line.

We first consider the 6-qubit ansatz with 18 parameters. In this case, we identify a saddle point characterized by a Hessian spectrum that contains several clearly negative eigenvalues, indicating the presence of well-defined unstable directions. The Hessian eigenvalues at this saddle point are provided in the Supplementary Information. Figure 6 shows the escape time plotted as a function of the effective noise strength $v=\sqrt{\eta /N_s}$ for different learning rates. Despite the increased system size, the data collapse onto a single curve when plotted against *v*, indicating that the escape dynamics are governed by the effective noise strength. A clear power-law scaling is observed, and a log-log fit yields

22 $$\begin{aligned} t_\textrm{esc} \propto v^{-0.3}, \end{aligned}$$

within the range of noise strengths considered. This behavior is qualitatively consistent with the 4-qubit results presented in Section Effect of measurement noise for the optimization dynamics, demonstrating that noise-assisted escape remains effective in this reduced-parameter regime, although the scaling exponent differs from the smaller system.

In contrast, for the 48-parameter ansatz, the escape dynamics show a markedly different behavior. Although saddle-like stationary points can still be identified, the number of unstable directions is very small compared to the total number of parameters, as revealed by the Hessian spectrum. In this regime, increasing the noise strength does not lead to a noticeable reduction in escape time. The escape time no longer exhibits a clear power-law dependence on the effective noise strength.

The above results indicate that noise-assisted escape in VQE critically depends on the local geometry of the loss landscape. When the stationary point possesses many unstable directions relative to the parameter dimension, as in the reduced-parameter regime, stochastic measurement noise can effectively drive escape, leading to power-law scaling of the escape time consistent with the continuous-time SDE analysis. In contrast, when the number of unstable directions is small, measurement noise alone is insufficient to induce efficient escape, and the power-law behavior observed in lower-dimensional or reduced-parameter settings breaks down.

In barren plateau regimes, it is known that gradients and Hessian eigenvalues become exponentially small with system size, and the number of unstable directions becomes negligible compared to the full parameter dimension. As a consequence, similarly to the highly parameterized 6-qubit ansatz studied here, stochastic measurement noise alone is not expected to induce noise-assisted escape, since there are insufficient unstable directions for noise to effectively drive the dynamics. We note that the present approach does not circumvent barren plateaus; rather, it clarifies the conditions under which measurement noise can facilitate transient escape dynamics in regimes where gradients and local curvatures remain observable.

## Conclusion

We analyzed the effect of measurement noise, i.e., the finite number of measurements, on the implementation of the gradient-based methods for VQEs. Our results demonstrate that the escape time from saddle points and an excited state follows a power-law scaling with respect to $\eta /N_s$, where $\eta$ is the learning rate and $N_s$ is the number of measurements. Through an analysis of SGD dynamics, we provide theoretical support for this scaling behavior, demonstrating how measurement noise facilitates escape from saddle points. This power-law scaling is accurately predicted by the continuous-time approximation of the discrete-time SGD using stochastic differential equations (SDEs). Despite the challenges in fully justifying the validity of this approximation due to subtle derivation issues, we find that the SDE model captures transient dynamics such as escape from saddle points with remarkable accuracy, even though it does not correctly describe long-time fluctuations in stationary states except for sufficiently small learning rates. Furthermore, we find that increasing the learning rate $\eta$ and decreasing the number of measurements $N_s$ have equivalent effects, both leading to a reduction in escape time. This equivalence, predicted by the continuous-time SDE, suggests that $\eta /N_s$ can be interpreted as the effective strength of measurement noise in VQE optimization. We find that the power-law behavior is also observed in another model (see the Supplementary Information).

The value of the scaling exponent itself is not universal, but depends on detailed properties of the model, such as the system size and circuit depth. Clarifying how these details determine the scaling exponent remains an open problem and is left for future studies.

Furthermore, extending our analysis to larger systems and different parameter regimes allows us to clarify the conditions under which noise-assisted escape is effective. In reduced-parameter regimes, where stationary points possess many unstable directions, measurement noise facilitates escape and the power-law scaling persists. In highly parameterized ansatzes, however, where unstable directions are scarce relative to the parameter dimension and effective escape pathways become difficult to identify, measurement noise does not noticeably accelerate escape, and the power-law behavior breaks down. We also note a qualitative difference between saddles and excited-state plateaus: in the latter, dynamics is almost frozen without noise, whereas a saddle can be escaped even in the absence of noise.

As we pointed out in Section Noise structure of SGD for VQE, the noise-covariance matrix is diagonal and has no remarkable structure. In contrast, it has been recognized that nontrivial structure of noise (i.e. its strong anisotropy due to the existence of off-diagonal matrix elements^48,54,58,61^ and its strong correlation with the loss function^61^) greatly helps optimization dynamics including the escape from saddles. Specifically, the absence of off-diagonal noise components suggests that introducing artificial structure in the noise covariance matrix could potentially accelerate the escape from local minima and saddle points, offering a promising avenue for enhancing the efficiency of VQE algorithms. Thus, introducing artificial structure into the noise covariance matrix could potentially accelerate the escape from local minima and saddle points.

In conclusion, our work underscores the importance of understanding the relationship between noise, learning rate, and measurement counts in optimizing the performance of VQE. Future studies should further explore how the choice of ansatz, parameter dimensionality, and more sophisticated noise models could deepen our understanding and lead to improvements in the performance of VQE and other variational quantum algorithms.

## Supplementary Information

Supplementary Information.

## Data Availability

The data that support the findings of this study are available from the corresponding author upon reasonable request.
