# Supplementary material for: Impact of measurement noise on escaping saddles in variational quantum algorithms
Source: Sci Rep. 2026 Feb 17;16:9390. doi: 10.1038/s41598-026-40123-3 (PMC13002861; doi:10.1038/s41598-026-40123-3)
Supplement: Supplementary file 1 — Supplementary Information. [file 41598_2026_40123_MOESM1_ESM.pdf]

Supplementary Information

1 Escaping from saddle points in XYZ model

We also consider the problem of finding the ground state of the one-dimensional (1D) XYZ Heisenberg model. The Hamiltonian is given by

$$H = \sum_{i=1}^{4} J_x X_i X_{i+1} + J_y Y_i Y_{i+1} + J_z Z_i Z_{i+1}. \tag{1}$$

Here, $J_x = 1.421$, $J_y = 1.288$, $J_z = 1.0$. The ansatz has the same settings as in Section 4 A of the main text, which is a typical hardware-efficient ansatz illustrated in Fig. 2. We measure the escape time from this saddle point. The parameters stay near a saddle point with the energy around -9.43. The eigenvalues of the Hessian at the saddle are (13.30, 10.31, 9.64, 8.38, 6.98, 4.87, 4.31, 3.23, 2.67, 2.54, 0.93, 0.37, 0.25, -0.16, -0.06, 0.052). The escape time is determined by the point in time when the energy $L(\theta^{(k)})$ falls below -9.7. Based on the continuous-time SDE discussed in Section 3, the noise strength of SGD is defined as $v = \sqrt{\eta/N_s}$. Similarly in Sections 3 and 4, the escape time from the saddle point for different values of η aligns along a straight line when plotted against $v = \sqrt{\eta/N_s}$.

The escape time behaves as

$$t_{\text{esc}} \propto v^{-1/2}. \tag{2}$$

Although the exponent of power differs from that in the isotropic Heisenberg model discussed in the main text, there is a power-law relation between escape time and noise strength.

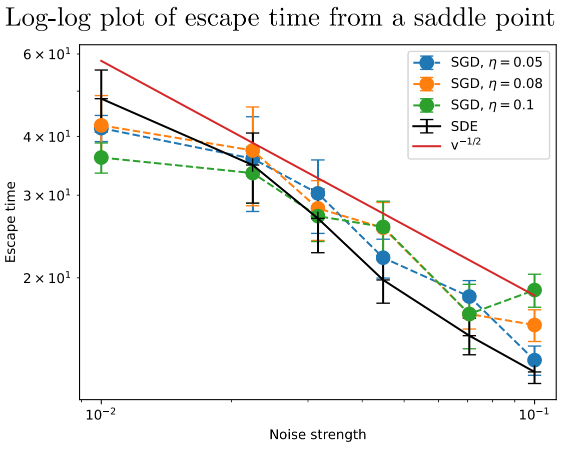

Supplementary Fig. 1: Escape time from a saddle point. We prepare 100 instances starting from the same initial condition, and compute the escape time averaged over them. The continuous-time SDE provides a good approximation even when the learning rate is relatively large.

2 Hessian eigenvalues at critical points

In this section, we report the eigenvalues of the Hessian matrices evaluated at the critical points discussed in the main text. These include the saddle points analyzed in Section 4 A, the excited-state solution discussed in Section 4 B, and the saddle points identified in the 6-qubit systems in Section 5.

For each critical point, the listed eigenvalues illustrate the local curvature of the loss landscape and, in particular, the presence or absence of unstable directions relevant to escape dynamics.

Saddle point (Fig. 4 (a)):
8.76, 7.77, 6.68, 5.99, 4.44, 4.15, 3.46, 1.97, 1.10, 0.358, 0.00850, 0.00397, 0.00, −0.0112, −0.0337, −0.118

Another saddle point (Fig. 4 (b)):
9.18, 7.51, 6.80, 6.54, 5.11, 3.98, 2.88, 1.66, 1.41, 0.45,0.22, −0.019, −0.0064, 0.0018, −0.0018, 0

Excited-state solution (Fig. 5):
7.84, 6.19, 5.58, 4.50, 3.95, 3.49, 1.36, 0.982, 0.574, 0.395, 0.272, 5.78×10^{-5}, 0.00, -3.20×10^{-7}, -1.54×10^{-5}, -8.28×10^{-5}

Saddle point in 6-qubit system with 18 parameters (Fig. 6):
10.65, 9.80, 6.99, 5.31, 5.14, 4.44, 3.98, 3.10, 2.79, 1.83, 1.35, 0.72, −0.25, 0.09, −0.11, −0.064, −0.0008, 0.

Saddle point in 6-qubit system with 48 parameters:
25.91, 16.76, 14.27, 12.65, 12.01, 11.39, 10.34, 10.34, 8.92, 8.78, 8.56, 8.13, 7.54, 7.28, 6.90, 6.84, 6.53, 6.03, 5.66, 5.65, 4.79, 4.70, 4.21, 3.99, 3.89, 3.11, 2.52, 2.50, 2.26, 1.80, 1.52, 1.43, 1.24, 1.09, 0.88, 0.77, 0.65, 0.55, −0.064, 0.37, 0.33, 0.28, 0.24, 0.19, 0.10, 0.065, 0.0070, 0.00085, −0.

Notably, in the reduced-parameter cases, the Hessian spectrum contains several negative eigenvalues. In contrast, for the highly parameterized 6-qubit ansatz, negative eigenvalues are rare when compared with the total number of parameters, as revealed by the Hessian spectrum.
